# Obesity Prevention in a City State: Lessons from New York City during the Bloomberg Administration

**DOI:** 10.3389/fpubh.2016.00060

**Published:** 2016-03-30

**Authors:** Paul M. Kelly, Anna Davies, Alexandra J. M. Greig, Karen K. Lee

**Affiliations:** ^1^ACT Health, Canberra, ACT, Australia; ^2^Australian National University Medical School, Canberra, ACT, Australia; ^3^Department of Health, Australian Government, Canberra, ACT, Australia; ^4^Dr. Karen Lee Health+Built Environment+Social Determinants Consulting, New York City, NY, USA; ^5^School of Public Health, University of Toronto, Toronto, ON, Canada; ^6^School of Public Health, University of Alberta, Edmonton, AB, Canada

**Keywords:** public policy, organizational innovation, environment design, active living, active design, food environment, regulation, leadership

## Abstract

**Objective:**

To illuminate the key components of multi-sector reform to address the obesogenic environment in New York City during the administration of Mayor Michael Bloomberg from 2002 to 2013, we conducted a case study consisting of interviews with and a critical analysis of the experiences of leading decision makers and implementers.

**Method:**

Key informant interviews (*N* = 41) conducted in 2014 were recorded, transcribed, coded, and thematically analyzed. Participants included officials from the Health Department and other New York City Government agencies, academics, civil society members, and private sector executives.

**Results:**

Participants described Mayor Bloomberg as a data-driven politician who wanted to improve the lives of New Yorkers. He appointed talented Commissioners and encouraged them and their staff to be bold, innovative, and collaborative. Multiple programs spanning multiple sectors, with varied approaches and targets, were supported. This study found that much of the work relied on loose coalitions across City Government, with single agencies responsible for their own agendas, some with health co-benefits. Many policies were implemented through non-legislative mechanisms such as executive orders and the Health Code. Despite support from academic and some civil society groups, strong lobbying from industry and an unfavorable media led to some reforms being modified, legally challenged or blocked completely, particularly food environment modifiers. In contrast, reforms of the physical environment were described as highly consultative across and outside government and resulted in slower but more sustained reform.

**Conclusion:**

The Bloomberg administration was a “window of opportunity” with the imprimatur of the executive to progress a long-term, multi-faceted obesity prevention strategy, which has successfully reversed childhood trends. Through the involvement of external researchers and the extensive use of empirical data from a wide range of participants, this study offers a unique insight into the ways in which this was achieved. While some of the aspects of the reforms in New York City are unique to that setting at that time, there are important lessons that are transferable to other urban settings. These include: strong and consistent leadership; a commitment to innovative approaches and cross-sectoral collaboration; and a context to support and encourage this approach.

## Introduction

Obesity is a complex public health problem requiring ­multi-pronged strategies to reverse long-term trends in rising global prevalence (1, 2). Governments have a role in developing and implementing policies and programs to address obesity, and this requires different agencies within government to act together (3).

During the Mayoral administration of Michael Bloomberg (2002–2013), New York City led the way globally in innovative, cross-sectoral policy interventions in obesity prevention. The wide-ranging reforms aimed to influence the food and the built environment to encourage lower caloric intake and more active lifestyles are outlined in Table 1. In general terms, the proposed reforms included those which provided incentives to industry or individuals, taxation and regulation, with a particular emphasis on the latter. Trans-unsaturated fatty acids (also known as partially hydrogenated oils or trans fats) were banned (4), calorie labeling was mandated at the point of sale (4), nutrition standards were mandated in City-run institutions (5), and a regulation limitation on the size of sugar-sweetened beverages (SSB; otherwise known as the “soda cap” or “portion cap”) was also proposed but eventually defeated in the courts (7). A tax on SSB was also proposed, but never enacted, through the New York State legislature (8). Incentives were provided to support diversification of the food supply in poor neighborhoods through supermarket construction (the FRESH initiative) and via mobile food carts, which offer only fresh produce (so called “green carts”) (9, 10). In the built environment, infrastructure provision and renewal acted to create incentives for active lifestyles, including the roll-out of integrated streets, cycle paths, a bike share program, and park facilities (11, 12). The use of the Active Design Guidelines, incorporating healthy concepts into design elements of both the internal and external built environment, has been mandated for use in major projects throughout the City (13).

**Table 1:** **Main legislative and regulatory reforms to support obesity prevention, New York City, 2002–2013**.

Policy framework	Setting	Intervention	Method	Year	Successfully introduced or enacted	Reference
Food environment	Farmers’ markets	Supplement to SNAP. $2 coupons for each $5 spent at farmers’ markets	Waiver request to USDA	2006	Yes	(14)
	Fast food restaurants	Trans fat ban	Health code	2007	Yes	(4)
	NYC facilities	All food purchased and served by city, including in schools, to meet nutrition standards	Executive order	2008	Yes	(8)
	Chain food service establishments	Calorie labeling	Health code	2008	Yes	(4)
	Street vendors (green carts)	New mobile food permits that only vend fresh fruit and vegetables in precincts with low consumption rates and lack of access	City council	2008	Yes	(9)
	Supermarkets	Zoning and tax incentives for supermarket creation and expansion (FRESH: Food Retail Expansion to Support Health)	Zoning text amendment and tax policies/city council	2009	Yes	(10, 15)
	Commercial buildings	Water bottle refilling stations	Plumbing code/city council	2009	Yes	(16)
	NYC schools	More stringent healthy food and beverage requirements than the 2008 executive order	NYC DOE chancellor regulation	2009	Yes, but modified by city council	(11, 17, 18)
	Commercial beverage industry	Sugar-sweetened beverage (SSB) tax	NY state legislature (proposed by State, supported by NYC)	2009 and 2010	No (proposed but not voted on as part of 2009 or 2010 budgets)	(8)
	SNAP vendors	Restrict use of SNAP benefits on SSB	Waiver request to USDA	2010	No (denied by USDA after hunger advocate/industry opposition)	(6)
	NYC schools	Water fountains	Federal law (healthy hunger free act)	2010	Yes	(19)
	New York City	Mandated annual report on nutrition environment	City council	2011	Yes	(20)
	Food service establishments regulated by DOHMH	Portion cap on SSB	Health code	2012	Yes, but overturned by courts	(7, 21)
	NYC licensed children’s camps	Nutrition guidelines, including guidelines on beverages served	Health code	2012	Yes	(22)
Physical environment	Cycling infrastructure	Cycle path expansion – on-road protected bike lanes and greenways	DOT program expansion	2007	Yes	(11)
	Cycling infrastructure	Street bicycle parking	DOT program expansion	2007	Yes	(11)
	Cycling infrastructure	Mandated indoor bicycle parking in new commercial and residential buildings; mandated entry into existing buildings with freight elevators	Zoning text amendment/city council	2009	Yes	(11, 23)
	Streets	DOT Street Design Manual for “complete streets”	DOT guideline for project approvals	2009	Yes	(24)
	Cycling infrastructure	Bicycle share program (citi-bike)	DOT-initiated PPP	2013	Yes	(12)
	NYC sponsored building projects	Routine use of Active Design Guidelines,^a^ Street Design Manual and LEED Pilot Credit “Design for Active Occupants”^b^ for new and major renovation design and construction projects	Executive order	2013	Yes	(12, 13, 25–29)
	NYC owned and leased buildings	Routine stairway access and use of stair prompt signage to promote stair use	Executive order	2013	Yes	(29)
	All buildings	Hold open device bill to facilitate stair visibility and use	City council^c^	2014	Yes	(30)
	All new buildings/major renovations	Public access stairway and stair prompt signage posting to facilitate stair use	City council^c^	2014	In committee	(30)
Food and physical environment	NYC permitted childcare centers	Nutrition, physical activity and screen time requirements	Health code	2007	Yes	(19)

*^a^Active Design Guidelines include a range of interventions in the outdoor and indoor physical environment to promote physical activity*.

*^b^Leadership in Energy and Environmental Design (LEED) green building rating system now has a pilot credit “Design for Active Occupants” created by NYC*.

*^c^Two bills introduced during Bloomberg administration, reintroduced in 2014 – one passed in 2014 and second bill pending*.

*NYC, City of New York; PPP, public–private partnership; SES, socio-economic status; SNAP, supplemental nutrition assistance program; SSB, sugar-sweetened beverages*.

Several papers have been published which outline the key elements and early achievements of the reforms (4, 5, 9, 11, 14, 19, 31–34). Less has been written about the way in which the reforms were successfully introduced or how these were perceived in the administration, the staff of various agencies across the City Government, by private industry, and by civil society (13, 14, 25, 33–37).

This paper does not aim to provide an analysis of all the individual policies and programs that occurred under the Bloomberg administration. Rather, it examines the experiences of many of the key decision makers and others involved in undertaking this work in New York City to illuminate the key components required to introduce and sustain whole of government reforms to address an obesogenic environment. While the paper is largely descriptive in nature, it will reflect on these experiences in the light of two theoretical frameworks relating to public policy (38) and public administration (39, 40).

### Aims and Research Questions

The study aimed to gain further understanding of the complex nature of cross-sectoral collaboration to achieve solutions for “wicked problems” such as obesity so as to assist public health practitioners in other settings to do likewise.

The specific research questions were:

1. What are the barriers and enablers to introducing and sustaining whole of government reforms to address the obesogenic environment?
2. How can public perceptions of the value of such reforms be assessed and/or influenced in relation to such reforms?
3. What does “success” look like? In particular, how can upstream changes best be measured to demonstrate early progress that could point to longer-term success?

### Theoretical Framework

Two theoretical frameworks informed this study. The first was Kingdon’s multiple streams theory of public policy, based on a series of interviews with people associated with the US Federal Government, is now over 30 years old, but remains useful when considering public health policy (38). Kingdon argues that three largely independent processes or “streams” operate in the public policy realm: the political environment, the identification and prioritization of problems, and the formulation of potential policy solutions to those problems. This theory proposes that the key to successful policy implementation is when all three streams intersect, usually for a brief period which he refers to as a “window of opportunity,” to allow problems to emerge, solutions to be recognized and for these to be politically supported. Kingdon further argues that key differences between the policy and the political realm include the varied pace of development, the dominant actors, and the way in which priorities and solutions are agreed upon. In politics, often unpredictable and rapid changes can lead to problem identification while potential solutions are often formulated by negotiation, which takes into account conflicting interests or by taking advantage of an often fleeting commonality of interests. Policy on the other hand, tends to be the realm of technical experts, is developed incrementally and relies on the diffusion of ideas and the gathering and testing of evidence. “Windows of opportunity” occur when varied interests coincide or certain events occur, which may include the political process itself (e.g., an election) or external incidents (e.g., media interest). The role of what Kingdon refers to as “policy entrepreneurs” is to recognize the intersection of the streams and thereby grasp these “windows of opportunity” to advance the policy agenda.

Moore has written extensively on the changing role of public service administrators and has argued that this role should not be restricted to legislative and regulatory functions nor to service delivery but rather to have a leadership role in creating what he has termed “public value” (39). His “strategic triangle” explains how the interaction of the authorizing environment, operational capacity, and public value can frame the work of public administration. The authorizing environment is what gives public officials the authority to act. While this is primarily the political authority that comes from elected officials and ultimately the electorate, it may also include legal authority and the legitimacy that comes from the support of individuals or organizations in the non-government sector. Operational capacity refers to the ability to perform the duties which are required to achieve certain objectives and these include financial resources, technical abilities, and people. Importantly, this concept is distinct from organizational capacity, and therefore includes the concepts of coproduction with partners inside or outside of government to achieve cobenefits. Public value describes both a process and an outcome and summarizes two distinct but related concepts. First, it refers to what the public thinks is being done by the public sector, and what in turn is their opinion on that performance. In short, their determination of whether the outcome is one that they desire and value. Second, the public’s appreciation that what is being done is something which is of inherent value in an uncertain future. This is particularly relevant to complex problems like the population health consequences of obesity, which will require sustained effort to remedy. In the discourse around the concept of public value, one of the important areas of debate is about who can and who should be deciding what is of value, and what role if any should non-elected public officials have in determining or even influencing these matters in a democratic system (40). These contested spaces highlight the crucial role of measurement of problems and their solutions to demonstrate the scale of the issue, what is being done and any change on the matter being addressed and to monitor public opinion of those government actions. Similar to Kingdon’s intersecting streams, strategies to create public value require the alignment of a convincing value proposition, sufficient and sustained political authorization, and operational feasibility (40).

## Materials and Methods

A case study methodology was employed which had two components: interviews with key informants and an extensive document review. The study protocol was developed in consultation with staff at New York City Department of Health and Mental Hygiene (DOHMH or Health Department).

Key informants, here termed participants, were purposively selected to gather information from those involved in obesity prevention and related activities during the Bloomberg administration. The aim was to include actors from both the health and non-health sectors, from within and outside of government and with a range of professional backgrounds and roles. All key informants were based in New York City. An initial list of 20 names was generated by DOHMH and then approached by the research team. A snowballing technique was used to identify additional potential participants.

The semi-structured interviews were guided by a list of suggested questions (see Supplementary Material). All interviews were conducted face-to-face by the lead investigator (PK). The key domains explored in the interviews were based on our *a priori* hypotheses and on the theoretical framework: the factors which motivated participant involvement, the role of leadership and governance in enabling policy reform, the assessment of public opinion of those reforms, the prospects for sustainability of the reforms, and perceptions of and ability to measure success of the reform agenda. Interviews were recorded and transcriptions were provided to participants to confirm accuracy. All data were de-identified and collated so that only general themes that emerge from the interviews are reported. Where quotes of participants have been used in the text, some minor editing has occurred to assist the reader to understand the context and in some cases to maintain confidentiality. The addition or substitution of words is indicated by the use of square brackets. Words or sentences that have been deleted from the quotes are indicated by the use of ellipses. Quotes have been rechecked by participants for accuracy and de-identified. Minor amendments to a small number of quotes were made by some participants to improve the clarity or grammar (41).

An iterative, cumulative process has been applied with regular reflections on the data collected. An initial list of descriptive themes was developed by PK based on the review of notes taken during the interviews. De-identified interview data were then coded by AD using the qualitative software ATLAS.ti version 7 (Scientific Software Development GmbH, Berlin, Germany, 2013). Inductive qualitative content analysis has been used to analyze the data that was then coded to reflect the themes that emerged from the interviews. Responses by multiple interviewees with their varied perspectives across and outside City Government and in different positions of power within their organizations were compared in the analysis of the transcripts of interview. In addition, interview data were triangulated with key documents including reports and other published materials as well as web-recordings of seminars and radio and print media interviews with key decision makers. These materials were reviewed by the principal investigator (PK) before, during, and after the interview phase to check the interpretation of findings and to see how closely the participant experience aligned with the public discourse of the period. What emerged was a more nuanced narrative and this is presented in the Section “Discussion.”

Ethical clearance was sought but deemed exempt by both the New York City DOHMH Institutional Review Board and the Australian Capital Territory (ACT) Health Human Research Ethics Committee. Despite this, written informed consent was still obtained from each participant.

## Results

Interviews were conducted in New York City from August to October 2014. Thirty-three face-to-face interviews were conducted with between one and four interviewees in attendance. This resulted in a total of 41 participants (Table 2) and almost 30 h of recorded material. The majority of the interviews (29/33, 88%) were with single participants. For the group interviews, two groups comprised of two participants, one of three and one of four. Answers to questions were regarded as individual responses and recorded and analyzed separately. There was only one interview where the identified key informant was administratively “outranked” by another person in the room, which may have influenced the responses given in this interview. Of those approached, one potential interviewee declined to be interviewed and two others were unavailable during the study period. There were no withdrawals from the study.

**Table 2:** **Sample characteristics**.

		Interviews	Participants
New York City government	DOHMH	7	8
	DPHO	3	5
	Other	7	10
Non-government	Health	4	4
	Non-health	2	2
Other	Academic	7	8
	Political	1	2
	Private sector	2	2
Total		33	41

*DOHMH, Department of Health and Mental Hygiene; DPHO, District Public Health Office; Private sector, design and construction industry*.

The resulting sample included participants from a wide range of sectors including 23 City Government officials (13 from health and the others from the departments of transport, design and construction, planning and parks), 8 academics, 6 employees of non-government organizations (NGO), two private-sector executives, a politician, and a political advisor. Several key decision makers and/or implementers of obesity prevention-related policies and programs were included. All interviewees, including NGOs and academics were either based in New York City or substantial components of their work were in New York City (two had offices in neighboring counties). The NGO employees were senior staff members of those organizations who were specifically and intimately involved with the work described. There were no lobbyists included in the sample. Several participants had moved to other positions, roles or sectors by the time of interview so their narratives quoted do not always apply to their job at time of interview, but rather to their role during the Bloomberg Administration.

While some new topics continued to emerge during interviews, saturation was reached on the major domains outlined in the interview schedule. The main theme that emerged from the data was the crucial leadership role which political and other influential actors had in encouraging or establishing, then fostering and building collaborative partnerships to achieve and sustain a public health agenda. Within this theme, there were several important enabling factors identified, including: a sophisticated understanding of obesity, its determinants and ways in which these could be influenced; a willingness to be bold and innovative; a willingness to collaborate; and a context which supported collaborative action and provided mechanisms for the adoption of innovative solutions.

### Leadership – Bold, Innovative, and Data Driven

All participants agreed that the authorizing environment for reform was strong. Perceptions on leadership of the obesity prevention effort during the Bloomberg administration varied, largely dependent on two factors. These were the role and position of the participant, and the participant’s opinion on the primacy of political commitment and authority versus bureaucratic leadership. In food environment reform, the encouragement to take on big health issues came from the Mayor, the thematic direction came from his two Health Commissioners, and the specific programmatic leaders were within the DOHMH. The Mayor was also a strong advocate of reform in the physical activity environment, but the other leadership roles were more dispersed, with important coordination work within the DOHMH together with prominent direction from other Commissioners and their staff as well as important contributions from the private sector.

There was universal agreement that Mayor Bloomberg was a “public health Mayor” and showed great commitment to improving the lives of New Yorkers. He was open to bold ideas to accomplish this.

Clearly Bloomberg knew public health; he had endowed a public health school at this point … I think he was really willing to push on this from a policy perspective … more than any other government official had. (Academic 1)

Essentially he *“appointed smart Commissioners [Department Heads] and got out of their way” (Academic 2, ex-DOHMH)* and supported their “*bold ideas*” in the public sphere when required. One participant suggested that he operated by “*pushing some things through*” to achieve his aims, and then being prepared to “*take the heat*” where these proved to be unpopular or controversial. As one participant described:

He wanted to do the big things and help out the people of the city of New York and he wasn’t using it as a stepping-stone for anything else and he didn’t have any other motives … So, if he saw something that he really believed was going to be good for New Yorkers and health was a damn important measure then he was going to do it because he felt that was what he got elected to do. (Academic 2)

Participants felt that he was able to be brave as he was not beholden to political donors, and lacked any further political ambitions. As one interviewee put it,

He came in as a multi-billionaire who could have done whatever he wanted, or [done] nothing (Academic 2)

He understood the complex data illustrating the issues and was guided by this in determining which policies he would support.

He wanted to see the data, he was big on data, he wanted to see the evidence … and if he believed that it would [work] he would be willing to support and he was really steadfast in his support of it. Notwithstanding the fact that it was particularly unpopular. (New York City Government [NYC Gov.], Health 1)

Mayor Bloomberg was prepared to use his executive powers to enact reforms to support the public health agenda (Table 1). Executive orders included mandating changed nutrition standards for food purchased by the City and the routine use of the Active Design Guidelines and related manuals for new and major renovation construction projects. Not all participants agreed with the Bloomberg approach to implementing initiatives, and voiced similar views to common critiques of his administration as being “top-down” and unresponsive to community perceptions particularly in poor neighborhoods.

I think the lack of community engagement is definitely a fair one. I think he was like: “well, here’s the data, don’t you get it?” (NYC Gov., Health 2)

[Bloomberg] didn’t engage the community or the larger city as a whole. He did dictate from on high (Academic 1)

This criticism of Bloomberg was not universally applicable, with participants also providing examples of substantial input from professional groups and civil society in the formulation and the implementation of some of the reforms, notably those associated with the Active Design Guidelines (26).

 … the [Fit] City Conferences [were] a platform that was created for public health professionals and design professionals to come together to have a dialogue … there were a whole series of workshops where design professionals were contributing ideas. That was a big part of working with our local American Institute of Architecture, so it was not just City Government, but included professional organizations and academics to diversify the dialogue. While it primarily involved professionals within the field at the very outset of the project, the Active [Design] Guidelines ultimately became a resource to help facilitate community outreach and to help provide community groups with a series of tools and strategies to advocate for healthy neighborhoods and cities. (NYC Gov., non-health 1)

Others suggested that this *“benign dictatorship”* (NYC Gov., Health 3) approach contributed to rapid implementation of Bloomberg’s broad reform agenda.

Could he have done a lot of things that he did if he did do it in that community oriented approach I think is an open question. (Academic 1)

In addition to Bloomberg, there were other leaders recognized by the participants, reflecting an element of distributed leadership in the reform agenda. This included the Commissioners (Department Heads appointed by the Mayor) during this period, including the two Health and the Design and Construction Commissioners, a number of specific directors working under the Commissioners, the Deputy Mayor, Mayoral staff, and some members of the City Council. As one participant explained:

[The Department of Design and Construction Commissioner] obviously had a huge amount of authority, which allowed us to proceed … and a huge capital works program here that made it meaningful and gave us traction. (NYC Gov., non-health 2)

Other leaders were key implementers in the bureaucracy from a range of agencies across the City Government. In this respect, leadership was about fostering and maintaining partnerships to expand upon and ultimately achieve the goals set by the Mayor and his Commissioners.

The [Department of Health Built Environment Director] I think did an amazing job … being a really, really committed and passionate person … to take it from an idea and good will and to create something … an enabling context if you will. (Academic 3, ex-DOHMH)

### Building and Sustaining Innovative “Coalitions of the Willing”

The importance of cross-agency collaboration was highlighted by many participants, especially in the formulation of the Active Design Guidelines. While the issue of obesity and the ways of getting to a solution was contested in the beginning, there were several strands of work where strong and lasting relationships and a willingness to understand others’ strengths and points of view flourished. Partnerships formed around common and often highly innovative ideas which came from within the DOHMH, other Departments, and from non-government sources including academia, civil society, and private industry. Importantly, and not surprisingly in such a contentious space, these “coalitions of the willing” were often matched and in some cases overwhelmed by “coalitions of the unwilling.” This was particularly prominent in relation to work in the broader food environment.

The complexity of the issue and the need for solutions to be implemented across many different sectors creates challenges when working to address obesity, and New York City was not immune to these challenges, particularly at the start of the work:

 … there wasn’t a cohesive coalition within the obesity agenda across the entire city. It was a very fragmented, multi-voiced and with disparate voices speaking all of the same end goal but not with the same policy or program ideas … it made the waters muddy … I think having a very cohesive coalition with a clear agenda could have probably helped. (NYC Gov., Health 4)

In fact, most participants agreed that the obesity prevention agenda for most of the Bloomberg period relied upon a loose coalition of largely un-connected groups within the City Government who had aligned agendas. These agendas included such diverse themes as: make New York City a better or more interesting place to live, work, move or eat; get the cars off the road through promotion of active travel options; sustain parks by diversifying and increasing their utility; and make streets safer and more user-friendly by reducing traffic and crime.

Active design was a great example … where all the agencies came together and all of them contributed and all of them took a stake in it. (Private sector)

In this scenario, multiple leaders were able to appreciate the health cobenefits which their proposals could bring, and to use those cobenefits to strengthen their collective arguments for funding, legislative reform, and community support and in some cases to engage in coproduction. The Health Department played a key role in being enthusiastic and vocal supporters of some reforms, helping to determine and publicize the health benefits rather than being the fundamental driver for some of these major reforms to the physical environment in the City. For example:

 … most of what the Department of Transport did, they did on their own but I could say we [DOHMH] acted as cheerleaders. We went to them to say “yes, this is not just [good] for transportation but also good for health”. (Academic 2)

Outside of the health sector, there were different motivations but many recognized the cobenefits, or had a very sophisticated understanding of the social and environmental determinants of health and their ability to influence this within their own discipline. This was particularly the case among participants who were key leaders in the active design initiatives. For some, this was a very obvious alignment of objectives.

 … when we started working on this active design work, as a group of designers, we were looking at it thinking, well, this is really not anything revolutionary, this is just basically good and responsible design, this is fundamentally what people should be doing anyway in designing and shaping their cities. (NYC Gov., non-health 1)

We saw a really great alignment with adding this new lens to talk about quality design that would have all these kinds of incredible public health benefits. (NYC Gov., non-health 1)

For others, the collaborative approach which brought together enthusiastic partners from such diverse disciplines was an innovative if not unique approach which led to new and effective ways of achieving these common aims.

I think it’s pretty rare … in … city governments around the world to have that kind of intensive collaboration on a project. We couldn’t have produced [the award-winning Active Design Guidelines] without the health evidence of our health colleagues. As health professionals they can provide the evidence and point to what the issues are but they do not necessarily know what the strategies are to help solve that … and that’s where the critical collaboration with design professionals comes in. (NYC Gov., non-health 1)

Most participants recalled a strong motivation to work through and address these challenges due to a commitment to the cause, a common interest in innovation, as well as a sense that what was being attempted was new and bold. Characteristics of this process included:

 … that was part of the ‘stickiness’ … we felt that we were making something brand new … that was really important. (NYC Gov., non-health 2)

 … a level of determination and a level of willingness to listen and adapt and understand different perspectives. (NYC Gov., non-health 1)

For the majority of the work, and particularly in the food environment reforms, participants described DOHMH as having led because they had the political permission to do something, and the data to support the agreed approach. However, Health Staff also recognized that the practical expertise to actually design and then implement the key population level reforms lay in other Departments and that they alone could not achieve the grand Mayoral vision of achieving a healthier city.

… [The Active Design work was] a really great example of cross-agency collaboration. Although there was a health outcome we were really concerned about, it was really the bread-and-butter work of some of the other agencies … We had really good people in those agencies who really agreed with us and really appreciated the convening, facilitating and supportive work of the [Health] Department. (Academic 3)

These reforms were recognized by participants as key contributions to creating an environment to support obesity prevention. Several participants described the Mayor’s encouragement of and investment in the revitalization and diversification of community open spaces and facilities throughout the city. He also encouraged the concept of “integrated streets,” which did not only concentrate on motor vehicle traffic.

So if you change that … little things: you’re moving people, not just cars, you include bikes, pedestrians and cars, right? … [the Department of Transport] also has lots of real estate so part of [the] job is … making spaces for people to linger in as well as walk or ride through. It isn’t just about mobility. (NYC Gov., non-health 3)

Opponents to some of the initiatives that Bloomberg implemented, or attempted to implement, however were described as persistent, persuasive, well-funded and well organized, and sometimes came from unanticipated directions.

The food industry, that’s soda companies, restaurants, grocery stores, bodegas, each have their own lobby group and are organized. Those [organizations] are mainly created by soda companies … the portion capping created one called New Yorkers for Beverage Choices, the tax one New Yorkers Against Food Taxes or something like that. They were completely transparent that they were front organizations and they weren’t really hiding much but it just showed they didn’t want to put the American Beverage Association or food companies name right up front. (Academic 2)

It’s incredible how much money the beverage industry can mobilize around the marketing of soda. We in public health have our little budgets [for social marketing], in the neighborhood of tens of thousands of dollars. The beverage industry, on the other hand, spent like $7 million in advertising in a couple of months … they had focus groups, they put together some really great materials. So there was big opposition. The marketing and media they can pay for takes up a lot of landscape … to shape people’s opinion. (NYC Gov., Health 5)

However, in the case of reforms to the food environment, all news appeared to be seen as good news, as this extensive public debate raised awareness throughout the community to an extent which is difficult to imagine standard social marketing alone could have achieved. A key example mentioned by several participants was the SSB portion cap proposal.

 … even the soda cap, we did not think it would be that shocking … you know, it was a bull’s-eye that we didn’t expect, including television shows talking about it and people doing scenes about it [in sit-coms]. (NYC Gov., Health 3)

Not all agreed with the approach, and this again was a perceived deficiency of the Bloomberg administration – the lack of interest, willingness or ability to create strong, sustained and widespread community support for some of the more controversial proposals.

I think the most telling critique of Bloomberg is that he didn’t try to build support for obesity control in New York’s poor communities and so it was possible for the food industry to characterize it as ‘nanny state’ work and as condescending and top down and it was possible for then the industry to either neutralize or enlist community leaders from black and Latino communities in opposing some of those Bloomberg initiatives … transformative changes in food policy and then obesity reduction will require community mobilization. (Academic 4)

Instead, in an effort to counter these negative voices, participants reported that the administration sought support from a strong “coalition of the willing”; those with an interest in obesity prevention outside of government – in civil society, in academia, in the media, and the political sphere – who were willing to stand up and support the proposals from the Mayor, his Commissioners and from the City Government. Consequently they actively sought to:

 … build collaborations with the university and with the New York City academic community to do something both from the knowledge side and also from the action side. (Academic 5)

 … you have to have other people because it is much more credible honestly to have other people doing it … ideally, we would have 40 scientists and nutritional leaders and other people standing behind us [for an announcement]. (NYC Gov., Health 1)

There was recognition both within the Health Department and outside of the need for such vocal coalitions to support the initiative and the crucial role of non-health and non-government actors, including academics, in confirming the facts and supporting the initiatives, and making statements in the public arena which were difficult or impossible for public officials.

 … ground softening, making people aware … highlighting the problem and ‘oh here is a really bold solution that nobody has ever done before but this is why it is important to do [it]’ … you have to have other people telling [this story] because it is much more credible … (NYC Gov., Health 1)

Later on in the process I learned how much influence, in terms of donations and money is involved from the industry and that kind of strengthened my thought that we have to voice our support a little stronger than we were typically comfortable with, just because the other side is so strong, based on very thin data. (Academic 5)

Similarly, civil society groups, other political actors outside of the City structure and even the private sector had their role as voices in favor of some of the reforms. In the relatively socio-economically disadvantaged Bronx Borough, for example, there were locally adapted programs especially in the nutrition environment where both the Borough President (an elected position with local leadership, advocacy and ceremonial powers) and a New York State Senator representing a Bronx district were prominent champions.

Throughout the City bureaucracy and in many non-government and academic circles, there was a willingness to be involved. This translated to a sense that anything was possible, that bold and innovative ideas were welcome, and that collaborative solutions were preferred. Within the Health Department in particular, this was a strong and consistent message, which came from the very top.

 … our Mayor who was a big supporter of public health issues, the Commissioner that cared about it, Federal funding [was received] to do more about it, was like ‘the golden era’ of alignments … that made it possible to get … innovative things on the table. (NYC Gov., Health 4)

Participants were driven by a passion to change New York City into a health promoting setting and many expressed pride in their world-leading work. Ultimately who took the credit and how this was achieved seemed less important to the participants than getting the job done.

Does it matter? I mean it’s sort of like religion. Does it matter your motivation as long as you get there? … if it’s a good thing, it’s all different paths to the same end. (NYC Gov., non-health 3)

### A Supportive Context to Achieve Success

In addition to a strong authorizing environment, collaboration across and outside the New York City government and a willingness to explore innovative solutions, all participants agreed that this ambitious reform agenda had a supportive environment that enabled it to flourish. Elements of this supportive context included: a willingness to use non-legislative regulatory powers such as the Board of Health and Mayoral Executive Orders; federal government funding for specific projects; the ability to collect, analyze, and report on health and other data; the capacity to plan and execute communications strategies; and dedicated legal support.

Many of the reforms, which were proposed in New York City during this period relied on the use of the non-legislative, regulatory powers available to the administration (see Table 1). The Board of Health provided a mechanism whereby regulations could be proposed and then debated by health experts in a public forum. This route was particularly prominent in relation to the reforms to the food environment.

So the fact that New York has this Board of Health with that capacity [to change laws separate from the City Council] … that changes the scope for a leader [in public health]. (Academic 6)

Another crucial influence was Federal Government and other external including philanthropic funding which not only compensated for City budget cuts caused by the Global Financial Crisis, but also focused activity because of the need to report on targets related to specific programs. This was particularly the case in the physical activity environment work where funding supported inter-sectoral engagement, coalition building, conferences, and training.

Well you know it [the Centers for Disease Control and Prevention grant] was certainly a big bolus of money but one has to appreciate at the same time there was a huge amount of local cuts. (Academic 3)

He [Mayor Bloomberg] even funded a lot of it through his … family money. (Academic 8)

Early in this period, and again influenced by Mayor Bloomberg’s background and interest in data, the importance of proving the effectiveness of the Health Department-led interventions was recognized and funded. Through this, the scope of data collection, analysis, and reporting was significantly strengthened. As one interviewee remarked:

In God we trust, all others bring data. (Academic 2)

 … tracking numbers was a central part of our religion in the Health Department … we were driven by numbers … [the other agencies were] much less data driven and their numbers were much more likely the process numbers. (Academic 2)

While solid data to support the need for action as well as the likely effectiveness of proposed approaches was important, there was a recognition that communicating these was a crucial component to success and that communication planning therefore needed to be integral from the beginning. Despite the pre-eminence of quantitative data, there was a recognition that stories are also important to counter negative perceptions.

It’s true that most politicians are driven by stories rather than data and we recognized that and tried to use the power of anecdote when we could … that wasn’t natural for me, so you know, the anecdote wars, we probably lost, but we won on the data wars! (Academic 2)

Similarly, many legal barriers or challenges were anticipated for some initiatives and internal legal counsel in the DOH was also involved from the very early planning stages.

Here we had a different sort of lawyer … who actually wanted to do policies and they were helpful rather than just coming in to say “don’t do it” because it’s too risky … when [the Health Commissioner] says “I want to do X”, [DOHMH legal counsel’s] job is not to say “no” but to tell me how to do X. (Academic 2)

As a result of this approach, there were many examples of reforms, which were successfullly introduced and sustained (such as the ban on trans fats, mandatory calorie labeling and the green cart expansion to areas of social and economic disadvantage), as well as some well-publicized failures (such as the aforementioned SSB portion cap proposal).

[DOHMH legal counsel] didn’t call it right every time, but we did a lot more than we would have done if it weren’t for [DOHMH legal counsel]. (Academic 2)

The method in which reform was achieved was often mutli-layered, and a range of ways of getting things done was explored, with some of these unique to the New York City context.

Obesity is obviously a multi-level disease but we therefore couldn’t think that things were going to change in obesity because of one or two strategies, so that idea of layering has become very important for me to begin to shift [the] social norm … for instance, sugar-sweetened beverages we looked to change things like that in a whole myriad of ways: … in early childcare centers, that was through the Health Code. We had a Mayoral executive [order] to change food standards for all city agencies … and that included no sugar-sweetened beverages … the Health Code [enforced] no sugar-sweetened beverages in day camps. (NYC Gov., Health 3)

The Health Department often laid the groundwork in gathering the evidence, stating the case for action and working collaboratively across government, and then Bloomberg acted using the powers available to him.

A big prelude to the executive order [to routinely integrate the use of the Active Design Guidelines on all city construction and design projects] was Health working individually with each of the city agencies to see what was feasible within their agencies. (NYC Gov., Health 6)

Within the City Government, while there were many examples of enthusiastic cooperation and collaboration, sometimes there was a need for strong governance to overcome the inertia that is inherent in any large bureaucracy. This lack of focus was not so much due to institutional reluctance but rather the presence of multiple competing priorities. In these circumstances, traction was created by the executive arm of government through the reinforcement of Mayoral priorities and at times by utilizing an event such as the release of a report to create opportunities to collaborate. However, at times, … *the most effective tactic was bludgeoning persistence* (academic 7, ex-Mayoral office).

Formal functional alignment of the City Government to address obesity, such as the New York City Obesity Taskforce (42) and other specific changes in governance structures occurred relatively late in the Bloomberg Administration. While some participants suggested that this was an attempt to widen the participation in reform, others saw this as a consolidation and an attempt to ensure sustainability.

Notwithstanding this concentrated effort, planning and innovative approaches and ideas, and all of the support which was available, including political support, many of the reforms did not succeed for a variety of reasons (see Table 1). However, several participants opined that the Bloomberg administration was highly successful in changing the conversation about obesity prevention at the population level.

 … the conversation that occurs because of what we’ve done … and when you do something controversial … you get a huge conversation … [which] begins to infiltrate the social norm. (NYC Gov., Health 3)

 … but then what happens is it starts getting into the vocabulary … if you say it enough and you keep doing it, it becomes … part of the vocabulary (NYC Gov., non-health 3)

Ultimately, it seems participants believed that success is best defined in terms of changing the cultural norm:

Healthy communities … that fundamentally is about norm change [which is] both cultural and policy oriented … ground-up individual, community and family leadership and … policies and programs that support and cultivate that. (Politician)

## Discussion

The crucial components of the ways in which a multi-agency complex intervention to address obesity in New York City was implemented during the Bloomberg administration, as perceived by central actors in the reforms, have been described. These can be summarized as the authority to act and leadership in acting, a commitment to collaborate and innovate and supportive structures to allow these to be enacted. This study is able to add a more complete picture than previous accounts due to: the time at which the study took place; the involvement of external researchers; and the extensive use of empirical data from a wide range of participants, triangulated with other published and electronic sources.

Kingdon’s three streams of problems, politics, and policies were clearly evident in the approach to obesity prevention in New York City during the Bloomberg administration (38). A problem, in this case rising rates of obesity and related chronic diseases, was recognized and brought to prominence resulting in a high degree of political support for doing something about this problem and a range of policy solutions were formulated, initiated, and sustained by “policy entrepreneurs” over a 12 year “window of opportunity” (4, 25, 31, 33, 34, 37).

In contrast to Kingdon’s theory, rather than pre-existing policy options being made available to match the political will to act, wide-ranging policies were developed by technical experts in the bureaucracy across the lifetime of the administration (25, 35, 36). To accomplish this task, two contrasting approaches emerged. In the food environment, policies were primarily and often relatively rapidly developed within the DOHMH utilizing the considerable regulatory powers available to that Department. In the physical activity environment, reforms were more likely to emerge either from outside the DOHMH or with a more deliberate and highly collaborative approach.

Moore’s strategic triangle is also relevant to explain the strengths and the weaknesses of the Bloomberg approach to obesity prevention (39, 40). Authority was clearly and consistently given from the mayor, but there were constant difficulties in other components of this crucial source of legitimacy to act – the city council, the courts of law, and the “court” of public opinion including the commercial sector. Operational capacity was clearly enhanced through deliberate decisions on the re-direction of internal funding and resources within health, the requirements to fulfill certain criteria of external funding sources, and in particular the innovative collaborations, which flourished across the City Government and with the non-government sector. Moore’s concepts of coproduction to achieve cobenefit was clearly evident in the Active Design Guidelines and related physical environmental reforms but less so in the reforms to the food environment. The one area in which the Bloomberg administration probably and crucially failed was in consistently building the public value proposition for the reform agenda.

A key question remains: what is unique to New York City and/or the Bloomberg administration and what is generalizable to other contexts? New York City, with a population of over 8.4 million people, is more populated than 39 of the 50 United States. While this does create challenges of scale and diversity, the substantially larger resource base and an innovative, well-trained workforce provides considerable comparative advantages over other cities in the US and elsewhere (43). So, while it is indeed feasible for health departments elsewhere to decide to employ lawyers, epidemiologists and communication specialists with specific tasks in obesity prevention initiatives, New York City has extensive resources with a consequent ability to carry out these vital supportive tasks in ways which may be beyond the capacity of smaller jurisdictions (4, 43). During the Bloomberg era, the City made the decision to allocate such resources to the task of addressing obesity.

### The Role of Leadership

The key attributes of public sector leadership have been extensively debated. This is particularly crucial when a government commits to tackling so called “wicked problems” like obesity where the issue, its underlying determinants, and the potential solutions are complex and sometimes contested (44, 45). In its simplest form, the role of a leader is to frame a vision and then inspire, enable, and empower others to pursue it (46). This is easily written but very difficult to consistently and sustainably put into practice in real world settings, particularly in the face of strong opposition from vested interests (2, 47).

This study confirms that Bloomberg possessed rather unique attributes for an elected official. These included a perceived lack of further political ambition beyond the New York City Mayoralty, non-reliance on campaign funding from external sources, a sustained interest in public health, and an appreciation of the pre-eminence of data to support decision making. The longevity of Bloomberg’s tenure and the stability, ability, and undoubted resolve of his Health and other Commissioners, as well as their directors and staff, was similarly unusual. Together, these elements of the administration allowed for a long-term view, a sustained focus, innovative solutions, and a systematic approach.

This strong, consistent, and prolonged political support for public health action was a feature of the Bloomberg period (36). The mayor provided a stable authorizing environment and stood by decisions, despite sustained campaigns against some of his initiatives via well-funded and highly visible campaigns by various business interests (e.g., sugar taxes, SSB portion cap), civil society (e.g., the schools and green carts), and in the media (42, 48, 49). An example was his continued support for controversial changes to motor vehicle access to Times Square during one of his re-election campaigns. He has been quoted as saying:

I don’t ask my Commissioners to do the right thing according to the political calendar. I ask them to do the right thing – period. (As quoted by former Department of Transport Commissioner Jeanette Sadik-Khan) (50)

However, as has been demonstrated in this paper, this political commitment at the highest level of government was not the only example of leadership during this public health “golden era” in New York City. Bloomberg appointed skilled and respected Commissioners who hired talented staff and they in turn were crucial in bringing the issues and potential solutions to him as well as implementing them. At this level, there was evidence of transformational leadership across multiple sectors who were prepared to set short and long-term goals, collaborate outside of their own portfolios, and dedicate substantial resources to get the job done (37).

### Innovative Solutions and Approaches to a “Wicked” Problem

The breadth of reforms and the truly cross-agency nature of the obesity-prevention agenda in New York City during the Bloomberg period were impressive. In Kingdon’s terms, there were a number of “policy entrepreneurs” within the bureaucracy during this period who, encouraged by the Mayor’s interest in the health of his constituents, actively pursued innovative policies and innovative ways of achieving them. Moore’s framework posits that the public sector manager should actively seek to improve the lives of the public which they serve by acting as a strategic decision maker with due regard to legitimacy, public value, and feasibility (40). This approach was similarly apparent during the Bloomberg administration (36).

There was an abundance of creativity to formulate new policies to influence the obesogenic environment at a population level (see Table 1). More importantly, there is strong evidence from this study that proves the existence of a governance system that allowed these ideas to be tested, and for data to be systematically collected and analyzed to further inform policy development. In essence, the Health Department did what they have called on others to do in understanding the context and successfully translating research into effective policy and programs (51). This is a well-recognized pre-requisite for successful implementation in the management literature (52). Examples included the use of simple signage to prompt stair-use (53, 54), and the various approaches used to foster changes in the food environment via commercial incentives such as FRESH, the green-cart initiatives, and calorie labeling (10, 15).

It was clear that one key enabling factor was the existence of the Health Code and the Board of Health. This non-legislative but regulatory function run by health experts is not unique to New York City (49). However, the innovative use of the Board of Health during this period, with a willingness to test the boundaries of the Board’s mandate in the prevention of chronic disease, was unique at the time. The trans fat ban and calorie labeling were successfully introduced through this mechanism, whereas the SSB portion cap was not. A key lesson here, which is more generalizable to other settings, is the importance of understanding the governance structures that are available and to use whatever means are feasible to achieve the best outcome.

This relates very much to the feasibility component of Moore’s ideal public administrator (40). Various commentaries on the tenure of Bloomberg’s first Health Commissioner, as reported in this paper and elsewhere, have pointed to the central role of measurement in policy discussions so as to prove that it was feasible to intervene, that intervention was likely to work and that this effect could be measured (35, 36).

A common view which was expressed by several participants in this study was that, as a major international city, New Yorkers perceive that they have a responsibility to lead the nation if not the world in multiple spheres. This resonated with Bloomberg’s view, shared by others across his administration, that attracting a dynamic, young, innovative demographic (50) was a key policy objective, in parallel with his agenda to increase life expectancy and tackle life-style related risk factors for chronic disease. This holistic view created many opportunities for coproduction to achieve cobenefits across multiple portfolios.

A criticism remains, one shared by some of the participants in this study, that these meso-level reforms did not address the underlying determinants of poverty, employment, and education among others, particularly in disadvantaged communities within the city (2, 33). Instead, New York City concentrated on what Backholder and others (55) have termed “agento-structural” interventions, those which aim to influence the environment but also rely on some level of individual action, essentially those which assist to make the healthy choice the easier choice (1, 36, 56). For example, the creation of open stairwells in the internal built environment and integrated streets in the wider physical environment provided opportunities for increased physical activity in the daily lives of New Yorkers, without mandating those choices. Similarly, in the food environment, calorie labeling was mandated to promote greater awareness, but food choice remains with the consumer.

Bloomberg has also been criticized, both by some participants in this study, and more widely in the public domain as being a “nanny,” that the reforms were all “top down” and that he concentrated on the “big end of town.” However, the motivations of detractors have been questioned (47, 48, 57, 58) and there is evidence of substantial community consultation with many of the public health initiatives. For example, the Department of Transport consulted widely on reforms, holding over 2,000 public meetings a year and even door-to-door consultations on local projects, for example bike lanes, bike-share infrastructure and traffic calming device installations (50). Similarly, the non-targeted nature of many of the policies and programs resulted in changes throughout the city, with arguably a greater public health benefit in low socio-economic neighborhoods (48).

### Collaborating Inside and Outside of Government

As has been confirmed in this study, the formation and sustaining of a wide-reaching “coalition of the willing” to support reform as well as developing strategies to deal with well-organized and vocal “coalitions of the un-willing” were prominent elements of the Bloomberg administration’s obesity prevention agenda. The strength and breadth of these coalitions varied over time and according to the particular component of the proposed reforms. Coalitions were within government and between government and various non-government actors and organizations. They were both formal, most notably in the physical environmental reforms and in particular in the work, which led to the formulation of the Active Design Guidelines, and informal, such as those related to the portion cap reforms where the academic sector were important allies and supporters.

Another important concept in coalition building, to which both Kingdon and Moore subscribe, is the role of coproduction to achieve cobenefits. In other words, collaborating to attain mutually satisfactory but sometimes widely dissimilar endpoints (25, 38, 59).

It has been suggested that coalitions of support are crucial to ensure implementation and sustainability of public health policies (59). Building and maintaining coalitions can be frustratingly time consuming, costly, and can be easily de-railed by a noisy counter-movement. In addition, coalitions of diverse sectors can lead to a “lowest common denominator” approach rather than what health experts may agree is best-practice (59, 60). One approach is to create a “neutral zone” to allow collaboration. In other words, to agree what can be agreed and work with that, while leaving contentious issues to one side, at least initially (59). As has been documented in this study, while compromise did occur, this was not a prominent aspect of obesity prevention during the Bloomberg administration where multiple novel and sometimes controversial reforms were pursued in parallel. The central role of “change agents,” Kingdon’s “policy entrepreneurs,” has been highlighted by others (59) and this role was particularly prominent in the writers of the Active Design Guidelines in New York City, both across government and outside government (26).

### “Coalitions of the Unwilling”

In New York City, during the Bloomberg administration, several of the most innovative and potentially effective reforms were blocked. Some of the organized opposition to reform appeared to be genuine concern by often quite local and spontaneously formed groups. However, much of the opposition to reform of the food environment was led, either overtly or covertly, by commercial interests. These groups were therefore not truly “grassroots” organizations arising from the community themselves, but rather “false grassroots” or “Astroturf” organizations being organized by external forces (36). This has strong echoes of the role of “big tobacco” in resisting smoking reform (2) and can be subtle (47) or blatant (8, 49, 57). Yanamadala and others (61) have shown that a high proportion of “community groups” opposed to food environment reform are actually funded by “big sugar,” that is, organizations with vested economic interests in the sugar industry (62).

The food stamp restriction failed because it relied on Federal agreement, which was withdrawn following pressure from advocacy groups including the National Association for the Advancement of Colored People and anti-hunger organizations (63). The sugar tax relied on agreement by the State legislature and was twice rejected after a concerted advocacy campaign by The American Beverage Association (ABA, the peak industry body for soda manufacturers), an Astroturf organization known as New Yorkers Against Unfair Taxes, various libertarian groups, and a former President of the American Dietetic Association (6, 34). The SSB portion cap proposal was vehemently opposed by industry groups (including New Yorkers for Beverage Choice which was initially funded by the ABA) (7).

Partly because of this well-organized opposition, nutrition reforms were the subject of extensive discussion before being enacted, and this often led to modifications. For example, the Board of Health received 38,000 written submissions (84% in favor) and a 90,530-signature petition in opposition, sponsored by the ABA. The regulation was passed by the Board of Health but was never enacted because of a legal challenge, again led by the ABA (7, 36).

Interestingly, the court decision to overturn the Board’s regulation, which was upheld on appeal, emphasized the principle of the separation of powers (arguing that the Board of Health can implement reforms but the City Council should lead on policy), and industrial fairness (not all purveyors of SSBs were affected equally) rather than any ruling related to undue government interference on personal freedom to choose (21).

### Contrasting Approaches to Food and Physical Environment Reforms

This study has described the two divergent paths taken in the obesity prevention agenda in New York City during the Bloomberg administration. In general, the nutrition reforms were more direct and top down. For many of the nutrition reforms, the “window of opportunity” was perceived to be short, there were potential mechanisms to enact reform via the Board of Health and so the Health Department essentially worked quickly and alone. Unfortunately, although causality cannot be directly attributed, the most contentious and most widely known of the reforms such as the soda cap and sugar tax proposals were unable to be sustained (see Table 1). This was in stark contrast to the active design work, which was described by participants as being characterized by a very collaborative approach across multiple city departments and the partners they engaged, with key coordination support from the Health Department. This appears to have resulted in much slower, incremental progress particularly in changes to the internal built environment, which was felt would be more sustainable in the long term. What is not clear is whether the success or lack thereof was due to the approach or the elements being addressed.

Contestability of how the problem is defined and what solutions were acceptable appears to be almost inevitable in nutritional reform to combat obesity (4, 49, 64). On the other hand, the existence of cobenefits to engender and sustain coalitions is clearer in active design. In the external environment, redesigned open spaces, with easy non-motorized vehicular access routes and adequate lighting increases the vibrancy of city life, decreases crime, increases social connectivity, decreases road traffic congestion and air pollution while increasing physical activity with a positive health benefit (25, 33). Similarly, facilitating the use of stairs rather than elevators and escalators in multi-story buildings can enhance environmental sustainability while increasing physical activity with health and productivity benefits (1, 13, 25).

The ultimately failed reforms in the nutritional environment (SSB portion cap, sugar taxes, food stamp restrictions) may have been due to a lack of consultation, but equally may have been due to the coordinated opposition of those who perceived these as threatening to their commercial interests. On the other hand, the sustained success of the active design agenda could be due to the highly collaborative and relatively slow-moving and iterative approach, but equally could be because there was strong consensus that what was being proposed had strong cobenefits to multiple sectors, and that what was being promoted as healthy design was really a subset or an extension of the predominant discourse in urban design, namely the building of attractive, usable, and sustainable urban environments on a human scale (25, 65).

It should be noted, however, that not all food-related initiatives resulted in conflict and opposition from the food industry. The Food Retail Expansion to Support Health (FRESH) Program was based on the creation of tax and zoning incentives for supermarket creation and expansion in underserved neighborhoods lacking in healthy food access. FRESH was created and implemented under the leadership of the Mayor’s Office working with City Planning, Economic Development, and the Health Department’s Built Environment Director who was also responsible for the active design work. The Program followed a similar trajectory to the active design work with strong support across multiple city departments and stakeholders and with a strong emphasis on the health and economic cobenefits of the initiative for the city, underserved populations, and supermarket operators. The FRESH Program, however, was not a regulatory food initiative but one based on the creation of incentives (10, 15, 25).

### The Bloomberg Legacy and Lessons Learnt

Much of the work of the Bloomberg administration remains in place under a new Mayor and some of the proposed reforms, for example in the building codes to encourage stair use, have now been enacted by the City Council. Despite the failure to influence SSB consumption through legislative means, there has been a statistically significant decrease in SSB consumption in both adults and children (64). This has led to participants feeling that, despite all the challenges, the work in this arena was worthwhile as it changed the conversation and social norms. There is furthermore emerging evidence of a stabilization and initial reversal of obesity rates in New York City’s children (64).

In addition, many of the Bloomberg reforms are being replicated and even expanded successfully in other settings. Trans-unsaturated fats have been banned nationally in the US, and this measure is also being considered elsewhere (66). Calorie labeling has been included in the Patient Protection and Affordable Care Act in 2010 (32, 67), alongside nutritional standards for children’s meals (68) and sugar taxes in Mexico and Berkeley, CA, USA (64). Active design principles are being adopted in multiple countries across several continents, with over 40 cities globally having worked with and/or working with the former New York City Health Department Built Environment Director as an advisor on obesity and chronic disease prevention issues (37).

### Limitations

This paper reports on a case study based on interviews of those who were involved in the obesity prevention reforms of the Bloomberg Administration, and therefore have a particular view of the events described here. The inclusion of outsiders with likely divergent views was out of scope of this study. Participants did, however, represent a wide range of perspectives, with those who work outside of DOHMH being well represented. The sample mainly included executive level staff and therefore decision makers and leaders rather than implementers. Not all of the key decision makers or early implementers were included in the list of participants. To address this limitation, analysis of peer reviewed publications by some of these individuals (4, 5), a recording of a public policy seminar (42), as well as key public interviews (50) was undertaken and analyzed in addition to the interview material.

## Conclusion

During the Bloomberg administration, the New York City Government may not have always got things right, but they had the permission and the dedication to attempt something bold and new, and there is much which can be learnt from their experiences. Some successes, such as the reversal of childhood obesity trends and SSB consumption did occur.

Multi-sectoral, multi-strategy approaches are generally recommended to address a wicked problem such as obesity. However, implementation of this method within existing governance structures in most settings is difficult. This period in New York City illustrates what can be achieved when there is sustained political will to act on the evidence-based advice of technical experts, even in the face of strong commercial and community opposition. Rather than rigid cross-agency structures, which are commonly formed to tackle wicked problems, the Bloomberg approach which consisted of a variety of multi-sectoral partnerships performing specific projects of work offers an alternative approach. This may be as simple as adding a health narrative to non-health interventions, which could be beneficial to obesity prevention, such as regulatory reforms or infrastructure to support active lifestyles.

Kingdon’s three streams theory (politics, problems, and policy) and Moore’s strategic triangle (authority, value and capacity) are useful in understanding how obesity prevention policy and practice operated in New York City during the Bloomberg administration. This period certainly represented a window of opportunity, and Bloomberg and others were undoubtedly policy entrepreneurs. The public value proposition of improving the health of New Yorkers was clearly articulated by politicians and within the bureaucracy and there was a concerted effort to increase capacity to act. However, the public value for individual reforms was highly contested in the public discourse, albeit with strong influence from the commercial sector. The failure to gain public support for many of the reforms could be seen as the most important limiter to the sustainability of the reforms of the Bloomberg period.

However, theoretical models have their limitations. What is clear from this study is the importance of the persistence and adaptability of public health practitioners who are tasked with applying theoretical frameworks and limited evidence of the effectiveness of interventions, often with scanty details on implementation, at the whole-population level. At the municipal level of government in particular, there are the additional challenges of often rapidly changing, complex circumstances at the interface between interventions and the conflicted views of multiple stakeholders.

The Bloomberg “golden era” for public health reform is remarkable due to the sustained effort and the multiplicity of interventions, in particular the willingness to work within the regulatory space. It remains to be seen if it is reproducible in other places and at other times and so there remains a need for further analytical research of similar and dissimilar governance models in different contexts.

## Author Contributions

PK conceived and designed the study, interviewed all the participants, and wrote the first draft of this manuscript. AD coded the interview data, assisted with the analysis and the drafting of the manuscript. AG assisted with study logistics, the interpretation of the data, and with the drafting of the manuscript. KL assisted during the recruitment of participants and contributed to the design of the study. KL, AD, and AG all critically revised the manuscript, and all authors have approved the final draft and agree to be accountable for all aspects of the work.

## Conflict of Interest Statement

KL was a key participant in the work described in this paper and was interviewed during the data collection phase. The remaining authors declare that the research was con­ducted in the absence of any commercial or financial relationships that could be construed as a potential conflict of interest.
