# Supplementary material for: Obesity Prevention in a City State: Lessons from New York City during the Bloomberg Administration
Source: Front Public Health. 2016 Mar 30;4:60. doi: 10.3389/fpubh.2016.00060 (PMC4812825; doi:10.3389/fpubh.2016.00060)
Supplement: Supplementary file 1 [file data_sheet_1.docx]

## SUPPLEMENTARY MATERIAL FOR MANUSCRIPT 178180_KELLY et al

## Semi-structured questionnaire for New York City participants, August-October, 2014.

Please note that this is the broad scope of questions. Some were not appropriate for some of the interviewees, and so the actual list of questions was a subset of this list.

1. Who are you?
   1. Age, gender, ethnicity, birth place, how long in NYC
   2. Professional and training background. Any in health, is so what?
2. What is your current role?
   1. Name of agency/organisation/etc
   2. Role in agency, length of service
   3. Key performance indicators, strategic direction etc of the agency and your position. Any health-related ones?
3. What is your personal/professional interest in health issues?
4. What do you understand about the term “health determinants”?
5. What is your understanding of the determinants of obesity? And of the health consequences of obesity? And of the societal consequences of high prevalence of obesity?
6. How would you assess the relative importance of population-wide approaches to combat obesity compared with individual approaches? (could give some specific examples here as prompts if needed)
7. What, if any, role have you had in the cross-agency approach to obesity prevention in NYC?
8. How/why did you become involved?
9. What proportion of you/your staff’s time is spent on this work? Has any of this work been “outsourced” to, for example non-government partner organisations? Has this changed over time?
10. Who are the “key players” (either people or agencies) in obesity prevention in New York City? Who leads/supports/follows/opposes or disengages?
11. What types of interaction do you have with others working in this field?
    1. Formal (eg. Regular meetings, teleconference, seminars, workshops)
    2. Informal (eg. Social gatherings, unscheduled meetings, “coffee”)
12. How would you describe the strength of that interaction?
13. What, if any, are the co-benefits that you and/or your agency have gained or envisage gaining over time from this collaboration on obesity prevention?
    1. Where does this sit in your list of priorities (high/medium/low…non existent)?
    2. Is it easy to see how this focus on obesity has changed the way you work?
    3. If any change, is this positive/negative?
14. What made this work easier (ie enabling factors), and what made it harder (ie barriers), from your perspective?
15. Were there any unintended (negative or positive) outcomes which resulted from your work on obesity?
16. How do you judge the strength of the obesity collaboration now (compared to the end of the previous administration)?
17. How do you see this work developing into the future? And your role/contribution to that work?
18. What does the public think about your work (and/or the wider work of the New York City Government) in relation to obesity?
    1. Have you ever tried to measure this support? If so how?
    2. Has there been any change in public opinion over time?
    3. What (if any) work have you been involved in to influence public opinion about the value of this work?
19. What does “success” look like from your perspective? In particular, how can upstream changes in the ways in which New Yorkers live best be measured to demonstrate early progress which could point to longer term success?
20. What existing (or novel) data collections have been used/could be used for quantitative analysis of change?
21. Have you employed any qualitative methods to assess knowledge, attitudes, perceptions or behavioural change in relation to obesity?
22. Do you have any documents, survey results, data analyses which you can share with me?
23. Any questions from you?
24. What are your contact details?
